# Real-time ultrasound enhances umbilical venous catheters placement in preterm newborns

**DOI:** 10.3389/fped.2025.1567586

**Published:** 2025-05-13

**Authors:** Francesca Cossovel, Francesca Galdo, Luca Ronfani, Laura Travan, Cristina Bibalo, Antonella Trappan

**Affiliations:** ^1^ Neonatal Intensive Care Unit, Institute of Child and Maternal Health, IRCSS Burlo Garofolo, Trieste, Italy; ^2^ Public Health Research Unit, Institute for Maternal and Child Health—IRCCS “Burlo Garofolo”, Trieste, Italy; ^3^ Community Medicine, University Hospital Giuliano Isontino, Gorizia, Italy

**Keywords:** umbilical catheters, ultrasound, position, catheter tip, radiography

## Abstract

**Objectives:**

the study aims to determine whether real-time ultrasound-guided umbilical venous catheter (UVC) insertion could enhance the accuracy of the procedure and reduce the number of x-ray, thereby minimizing radiation exposure of the newborn.

**Methods:**

A pre-post study was conducted, comparing outcomes before and after the introduction of ultrasound during catheter placement. All included newborns required placement of the UVC. In the study population (interventional group) the catheter tip was visualized using both ultrasound and radiography, whereas in the in historical cohort (control group) the catheter tip was visualized solely through radiography. Exclusion criteria included hemodynamic instability, known vascular malformations and major congenital malformations.

**Results:**

During the study period, 33 eligible neonates underwent tip navigation and were enrolled, all of whom had a centrally-placed UVC. Additionally, 36 newborns were retrospectively identified as control group. The median gestational ages were 34 weeks and 33 weeks respectively for the interventional and control group (*p* 0.74). The median birth weights were 2,087 (1,400–3,220) g and 1,966 (1,489–2,695) g respectively for the interventional and control group (*p* 0.67). The catheter was correctly positioned at first attempt in 29/33 (87%) patients with US guidance and 17/36 (47%) in the control group (*p* < 0.001). The mean numbers of x-rays taken were 1.1 and 1.8 respectively for the interventional and control group (*p* < 0.001). In the control group, the mean number of antero-posterior thoracoabdominal radiograph (TAR) ranged from 1 to 3 for each patient.

**Conclusion:**

In conclusion, ultrasound could be the standard of care for umbilical catheter placement, ensuring accurate vessel assessment and real-time visualization. Despite our limited sample, our findings highlight its superior precision and safety, reducing complications and the need for radiography. Integrating ultrasound into daily neonatal practice can improve catheter placement and patient outcomes.

## Introduction

The umbilical venous catheter (UVC) is the most commonly used venous access in Neonatal Intensive Care Unit (NICU) and can be placed in premature infants or full-term infants. It is crucial in managing both critical and non-critical patients for the administration of parenteral nutrition, hypertonic solutions, medications, transfusions, and for providing emergency venous access. The placement of a UVC is a procedure performed by adequately trained personnel, and it is essential to confirm its correct placement to avoid serious complications and administer infusions with higher osmolarity. Common complications include cardiac arrhythmias, thrombosis, myocardial perforation, pericardial effusion, or tamponade if the tip is intracardiac. Alternatively, the presence of the UVC in the portal vein can cause thrombosis, portal hypertension, or hepatic necrosis (1).

Typically, the UVC is inserted to a predetermined length from the umbilicus and tip position is then assessed by antero-posterior thoracoabdominal radiograph (TAR). If the catheter is not correctly placed, it must be repositioned and checked again with x-rays involving additional handling of the central line and of the neonate, radiation exposure, and treatment delays. Various methods to calculate the UVC insertion length have been studied, including Dunn's shoulder umbilical length graph (2) and Shukla's formula based on birth weight (3). However, Lean et al. (4) demonstrated that, even when the best formula is used, almost half of umbilical venous catheters inserted may require manipulation to achieve a safe position.

The ideal UVC tip position to minimize complications is outside the heart, at the junction of inferior vena cava (IVC) and right atrium (RA). However, this is not easily translated into a well defined radiological landmark. Traditionally, UVC tip position is evaluated in relation to vertebral bodies, cardiac silhouette and diaphragm. The catheter should be at the diaphragm level or slightly above or between the vertebral bodies T8 and T9 (5) or at the cavo-atrial junction obtained by extrapolating the curve of the RA medial border up to its intersection with the IVC or with the right border of the vertebral bodies (6).

Furthermore, radiography exposes the patient to radiation, even multiple times in case of repositioning, and requires the use of multiple resources (radiology technician, radiologist) that are not always readily available. Conversely, echocardiography allows direct visualization of the cavo-atrial junction, making this technique superior to chest radiography in determining the position of the UVC tip. Moreover, real-time ultrasound use during umbilical vein catheterization is a promising technique for rapid and precise catheter placement (7–10). Compared to traditional methods, ultrasound significantly reduces malpositioning, complications, and the need for repositioning attempts. Additionally, it enhances the effectiveness of facilitation maneuvers ensuring optimal placement (11). Ultrasound for tip navigation, therefore, due to its rapid execution, non-invasiveness, and accuracy, is a technique rapidly spreading within our Neonatal Intensive Care Units (12). There is increasing literature highlighting the qualities of this method, which, among other things, requires operators with simple basic training.

Our study aims to verify if real-time ultrasound-guided UVC insertion could increase the accuracy of the procedure itself and limit the number of x-rays, thus reducing the radiation exposure of the newborn.

## Methods

This is a pre-post study conducted in IRCCS Burlo Garofolo, Italy, before and after the introduction of ultrasound during catheter placement. The study protocol was approved by the Bioethical Committee.

The study population (interventional group) included infants born between October 2019 and September 2020 who required placement of the umbilical venous catheter for therapeutic reasons. In the Interventional group, the catheter tip was visualized both with ultrasound and radiography.

Our historical cohort (control group) included all neonates born at our tertiary-level hospital, Institute for Maternal and Child Health IRCCS Burlo Garofolo of Trieste, Italy, between February 2018 and March 2019 who required placement of the umbilical venous catheter for therapeutic reasons. In the control group, the catheter tip was visualized only with radiography.

All the newborns admitted to our NICU were enrolled after obtaining informed written consent from parents. Exclusion criteria included hemodynamic instability, known vascular malformations and major congenital malformations.

Umbilical catheters were inserted under sterile conditions by the clinical team (neonatologists and neonatal fellows), with their length predetermined using Shukla's formula or Dunn's graph based on shoulder-umbilicus length, while the gauge was selected according to the patient's weight. In critically ill neonates requiring multiple incompatible therapies, a double-lumen catheter was chosen; otherwise, a single-lumen catheter was placed.

Ultrasound scans were performed by trained neonatologists using a Logiq E9 Ultrasound Unit (GE Healthcare) equipped with S4–10 microconvex. The US operator stayed by the bedside and put the probe under the sterile field either immediately after initial catheter insertion or later, when the predefined depth was almost reached without feeling any resistance, indicating a good chance of having bypassed the ductus venosus. In order to visualize the pathway of the catheter and especially the junction between IVC and RA, scans were performed through a subcostal view. When necessary, a small volume of saline was injected so that turbulence could better identify the catheter tip. The neonatologists who performed the ultrasound scans underwent training in pediatric cardiology to learn the correct ultrasound window for visualizing the atriocaval junction. Subsequently, in the NICU, they performed over 15 scans on different patients with an umbilical venous catheter to assess the correct positioning of the tip. Throughout the study, the trained neonatologists were supervised by an experienced neonatologist in echocardiography, who confirmed the correct catheter placement.

Iconographic documentation (photography or video) was recorded for each patient.

Once the position was considered optimal, the catheter was secured and TAR taken.

The primary outcome was the difference in the rate of success of correct UVC placement at first attempt (defined as correct placement after the first x-ray, with no need of repositioning) between the two groups. Newborn with low-lying UVC were excluded from the analysis.

The secondary outcome was the difference in the number of radiographs obtained in the two groups.

Categorical variables are presented as numbers and percentages, and continuous variables as medians and interquartile ranges (IQR), given the non-normal distribution of data. Differences between groups were evaluated with the Chi-square test (or Fisher's exact test when appropriate) for categorical variables and with the Mann–Whitney test for continuous variables since a non-normal distribution of data was found both visually and with the Kolmogorov–Smirnov test. Differences with *p*-value <0.05 were considered statistically significant. The sample size was predetermined. Preliminary data showed in the control population a first-attempt success rate (primary outcome of the study) of 30%. Assuming in the intervention group a first-attempt success rate of 70%, it was estimated the enrollment of 30 subjects per group to conduct the study (60 in all), setting alpha at 0.05% and beta at 0.20.

## Results

During the study period, among the neonates who underwent tip navigation 33 were enrolled and all of them had a centrally-placed UVC. 36 newborns were retrospectively identified as Control group.

The median gestational ages were 34 weeks and 33 weeks respectively for the interventional and control group (*p* 0.74). The median birth weights were 2,087 (1,400–3,220) g and 1,966 (1,489–2,695) g respectively for the interventional and control group (*p* 0.67). Table 1 shows the baseline characteristics of the patients in the 2 groups (Table 1).

**Table 1:** Baseline characteristics of the patients in the interventional and control group.

Characteristics of the patients	Interventional group (*n* = 33)	Control group (*n* = 36)	*p*
Male, *n* (%)	17 (51.5%)	20 (55.6%)	0.74
Prematurity, *n* (%)	21 (63.6%)	24 (66.7%)	0.79
Birthweight (grams), median (IQR)	2,087 (1,400–3,220)	1,966 (1,489–2,695)	0.67
Neonates <1,500 g, *n* (%)	10 (30.3%)	10 (27.8%)	0.82
Gestational age (weeks), median (IQR)	34 (31–39)	33 (32–38)	0.73
Day of insertion, median (IQR)	3 (1–4)	2 (0–4)	0.32

**Figure 1:**
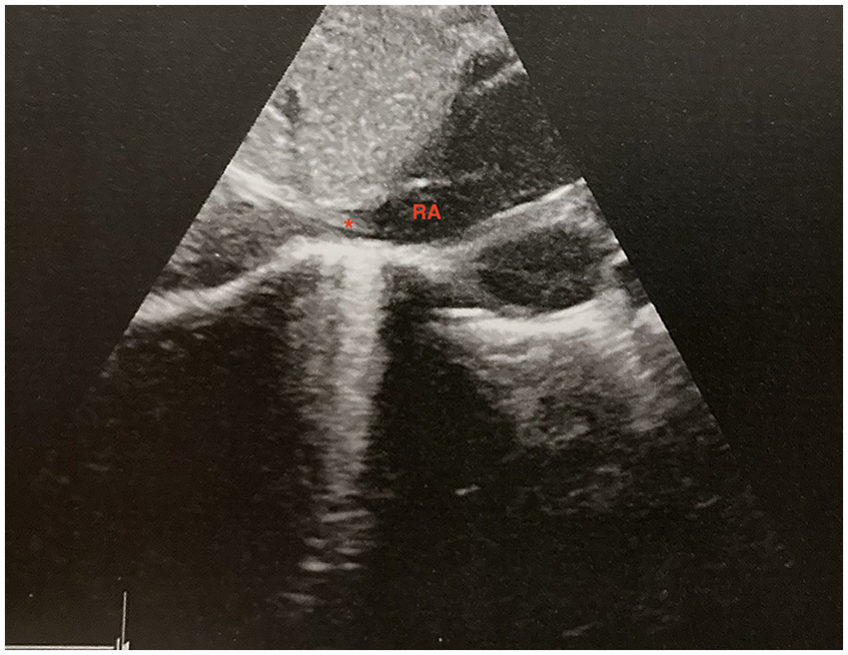
Ultrasound image of the correct placement of the umbilical venous catheter during a saline solution flush. RA, right atrium. * Catheter's tip.

The catheter was correctly positioned at first attempt in 29/33 (87%) patients with US guidance and 17/36 (47%) in the control group (*p* < 0.001). Figure 1 shows a representative ultrasound image of a correctly placed catheter. In the US group, in the 4 neonates where the CVO was not visualized, the ultrasound window was not adequate due to pulmonary barrier or excessive gastric bubble. The mean numbers of x-rays taken were 1.1 and 1.8 respectively for the interventional and control group (*p* < 0.001). In the control group, the mean number of TAR ranged from 1 to 3 for each patient.

## Discussion

US-guided UVC placement improves the success rate of the procedure, defined as correct catheter tip placement, reduces the number of catheter manipulations, and decreases the number of radiographs required.

Proper catheter placement is crucial in an emergency setting such as in a NICU because it ensures safe vascular access during emergencies and allows the administration of life-saving therapies. If the catheter is properly positioned, it prevents the onset of complications such as cardiac arrhythmias, thrombosis, myocardial perforation, pericardial effusion, and tamponade (1). Several studies demonstrated that ultrasound enables faster and more accurate catheter placement (1, 13, 14) than radiography. Fleming et al. highlighted that using US-guided UVC placement saves of over an hour of time for the procedure. This result is extremely important when considering NICU patients who are at risk of severe hypoglycaemia and early sepsis. Having rapid and secure vascular access allows for the prompt administration of parenteral nutrition and antibiotics.

The speed of the sonographic technique is attributed to the reduced involvement of resources (radiology technicians, radiologists, computer systems, machinery). For the same reason, this technique also proves to be less costly (15).

Another benefit of US-guided UVC placement is the reduction in catheter manipulations, as confirmed by Fleming. Each catheter manipulation increases the risk of trauma, infection, and thrombosis (13, 11). Reducing manipulations also decreases the need for repeat radiographs and, consequently, radiation exposure. In our study, ultrasound reduced the use of radiographs by 40% (*p* < 0.001), despite our protocol stipulating that radiographs be performed in all patients in the interventional group. Other studies with smaller sample sizes than ours have also confirmed this result (1, 13, 14).

The limitations of our study are the study design and the sample size. We opted for a pre-post study design because, when the ultrasound machine was introduced in the department, it was preferred to also maintain radiographic control, as the staff was more confident with radiographic interpretation. The sample size was limited but it was calculated before conducting the study to ensure statistically significant; moreover, in the literature, studies rarely present numbers larger than ours.

In conclusion, ultrasound guidance enables accurate vessel assessment and real-time catheter visualization (16). Its routine application improves precision, decreases complications, and reduces reliance on radiography, which should be reserved for cases where ultrasound alone proves insufficient. Although our study was conducted on a limited sample, with a low representation of extremely low birth weight preterm neonates, our findings highlight the superior sensitivity, effectiveness, and safety of ultrasound in the general neonatal population. Further studies specifically targeting ELBW infants are needed to confirm the safety and feasibility of ultrasound-guided UVC placement in this particularly fragile group. Integrating ultrasound into daily neonatal practice can significantly improve catheter placement and overall patient outcomes.

## Data Availability

The raw data supporting the conclusions of this article will be made available by the authors, without undue reservation.
